# Hem-o-Lok clip migration into renal pelvis and stone formation as a long-term complication following laparoscopic pyelolithotomy: a case report and literature review

**DOI:** 10.1186/s12894-022-01015-6

**Published:** 2022-04-19

**Authors:** Hui Zhou, Yihuan Li, Guangjie Li, Guobiao Liang, Zeju Zhao, Xu Luo, Shulian Chen

**Affiliations:** Department of Urology, Affiliated Hospital of Zunyi Medical University, 149 Dalian Road, Zunyi, 563000 Guizhou China

**Keywords:** Laparoscopic pyelolithotomy, Hem-o-Lok clip migration, Renal stone, Percutaneous nephrolithotomy, Case report

## Abstract

**Background:**

Hem-o-Lok clips (HOLCs) are widely used in minimal access urological operations due to the advantage of vascular control and suture stabilization. In rare cases, however, they can develop problems themselves. Migration of HOLCs into the collecting system is a fairly rare complication after laparoscopic pyelolithotomy. To date, only two cases were reported in the literature.

**Case presentation:**

This article describes a case of 51-year-old man with a complaint of left flank pain. He had a medical history of ipsilateral retroperitoneal laparoscopic pyelolithotomy at another hospital 8 years ago. Non-contrast CT scan demonstrated a renal stone in the left ureteropelvic junction complicated by mild hydronephrosis. A straight foreign body was found near the renal pelvis, with part of it wedging into renal pelvic wall. A percutaneous nephrolithotomy (PNL) was performed for this patient. After some fragmentation, a HOLC was found in the kernel of the stone. With an alligator plier, the clip was totally removed out of the collecting system. The postoperative period and follow-up were uneventful.

**Conclusions:**

HOLC migration into renal pelvis is a rare complication following laparoscopic pyelolithotomy. It could act as nidus for stone formation under extended exposure to urine. Using HOLCs to stabilize the anastomotic suture near renal pelvis should be avoided to prevent this complication. Instead, knotting is a better choice under such condition. The secondary calculi and dislodged HOLCs can be removed through PNL by an alligator plier after laser lithotripsy.

## Background

To date, Hem-o-Lok clips (HOLCs) have become an important instrument in minimally invasive surgery, as a result of the capacity of vascular control and suture stabilization [1]. They are also widely used in minimal access urological operations, including robotic or laparoscopic radical cystectomy, prostatectomy, nephrectomy or partial nephrectomy. However, in rare cases, they can develop complications themselves, especially in reconstructive procedures [2]. This report describes a rare case of renal stone formed by a migrated HOLC 8 years following the initial retroperitoneal laparoscopic pyelolithotomy.

## Case presentation

A 51-year-old man with a complaint of left flank pain was admitted to our department. He had a medical history of ipsilateral retroperitoneal laparoscopic pyelolithotomy at another hospital 8 years ago. By reviewing the medical records, the volume of removed calculus was about 22 mm × 18 mm in size. A Hem-o-Lok clip was used to stabilise the running anastomotic suture of renal pelvis. The patient recovered well after the surgery. A KUB plain 8 days after the operation confirmed no residual stone fragment. The ureteral stent was removed 4 weeks postoperatively.

**Fig. 1:**
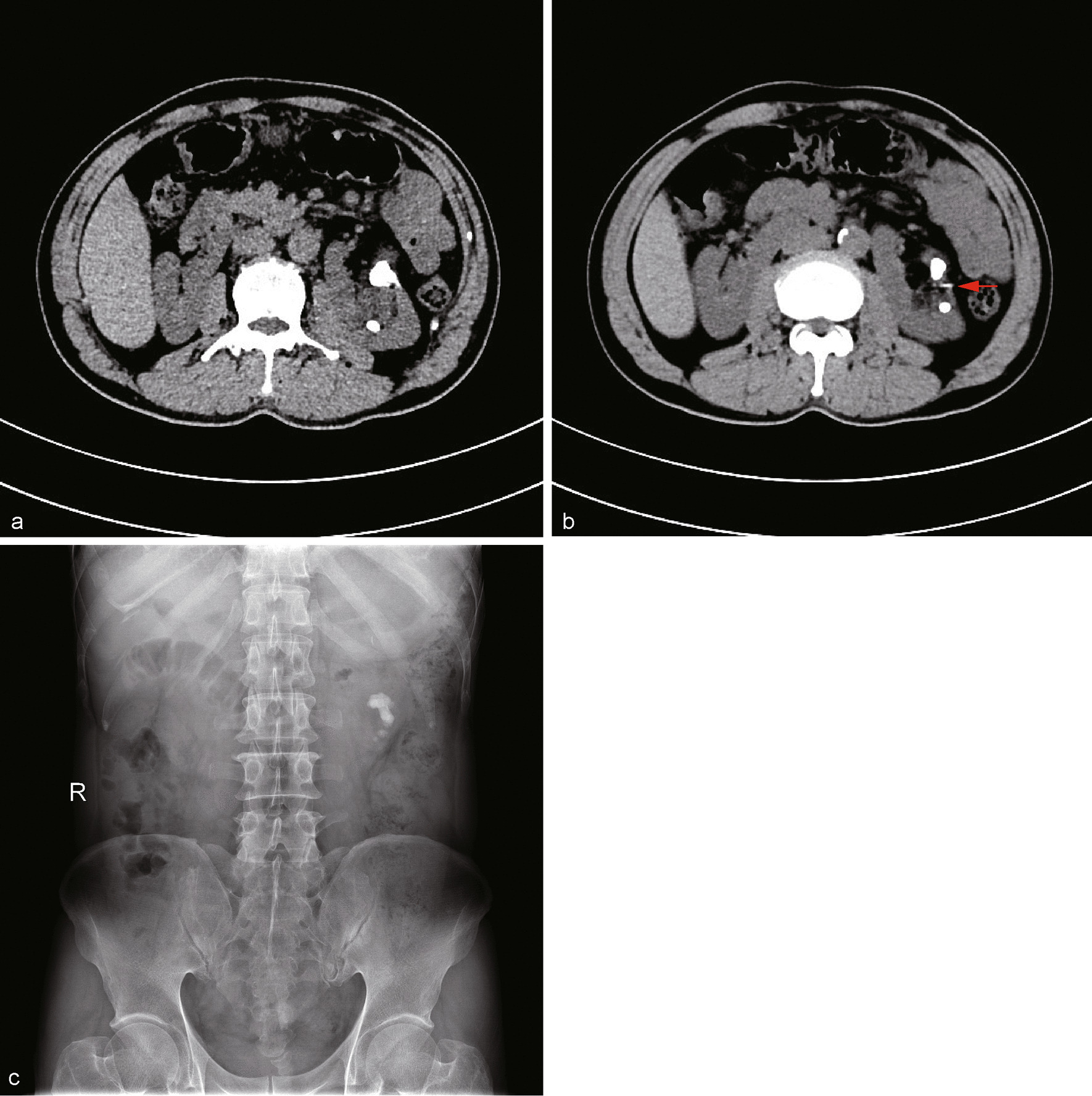
Preoperative Imaging. **a** CT image showed left renal stones with mild hydronephrosis. **b** CT scan revealed part of the HOLC imbedded into renal pelvic wall (red arrow). **c** A KUB plain displayed the left kidney stones about 25 × 20 mm in size

**Fig. 2:**
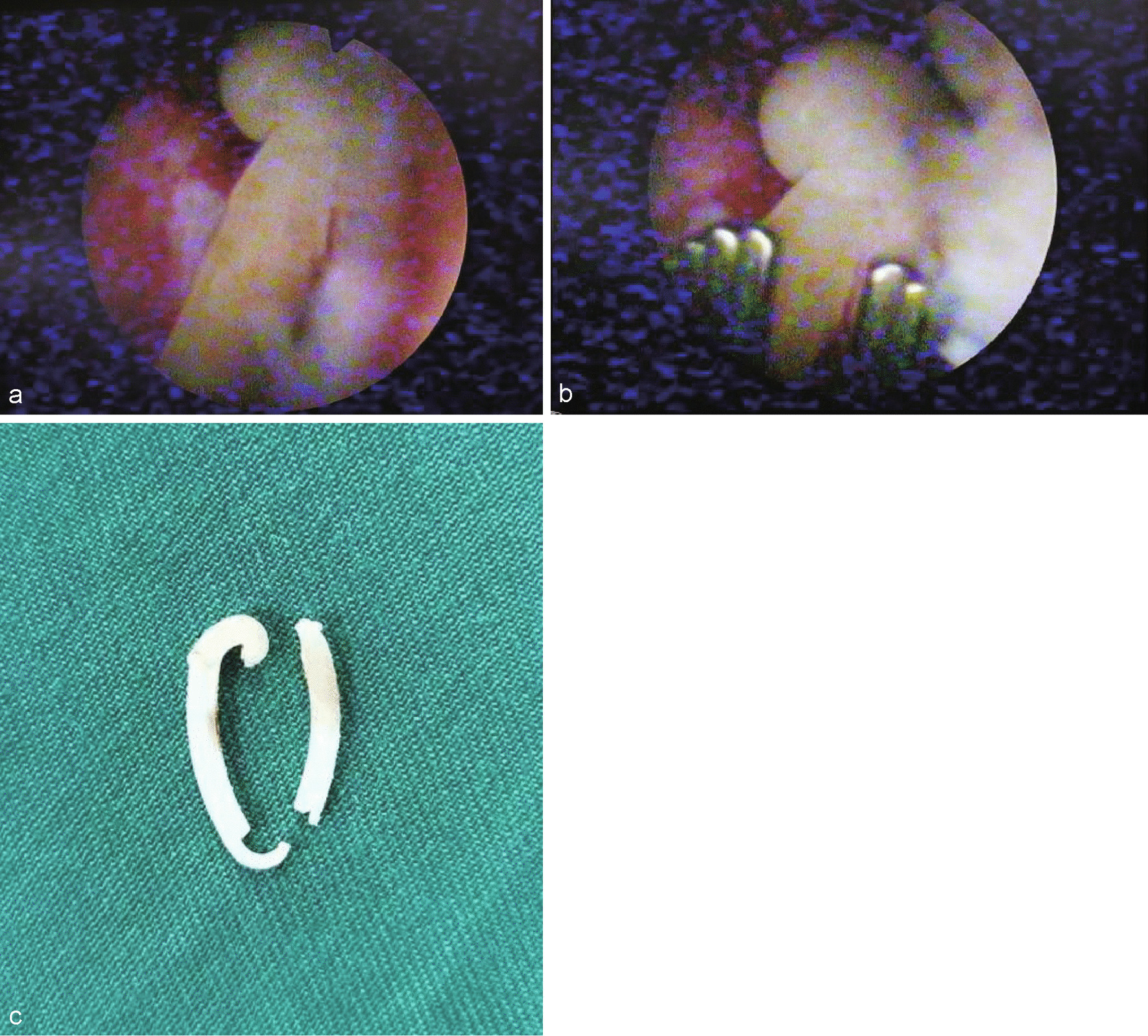
Intraoperative Images. **a** A nephroscopy found a HOLC after comminuting the stone. **b** The clip was removed by an alligator plier. **c** The removed clip

Current non-contrast CT scan demonstrated a renal stone in the left ureteropelvic junction complicated by mild hydronephrosis (Fig. 1a). A straight foreign body was found near the renal pelvis, with part of it wedging into renal pelvic wall (Fig. 1b). A KUB plain displayed a renal calculus about 25 × 20 mm in size. A percutaneous nephrolithotomy was planned for this patient. Under general anesthesia, in prone position, a 22Fr access was established under the guidance of ultrasound. With a 20Fr nephroscope, the stone was visualized. Via the working channel of nephroscope, using an ultrasound probe, lithotripsy was performed. After some fragmentation, a HOLC was found in the kernel of the stone (Fig. 2a). Removal of this foreign body was troublesome, as one-third of it tightly embedded into the wall of renal pelvis. With an alligator plier, the clip was totally removed out of the collecting system (Fig. 2b, c). The postoperative period was uneventful and the patient was discharged 7 days after the operation. There was no stone recurrence at 6 months of follow-up.

## Discussion and conclusions

If foreign bodies, like metallic coils, surgical staples, titanium and Hem-o-Lok clips, enter the collecting system, they can serve as a nidus for stone formation under extended exposure to urine [3]. With the wide use of HOLCs in laparoscopic operations, relevant complications step into the vision of urologists. Herein, we reported a rare case of renal stone formed by a migrated HOLC 8 years after initial laparoscopic pyelolithotomy.

In this patient, we found a straight foreign body near the renal pelvis preoperatively (showed in Fig. 1b), but wasn’t aware of the issue of clip migration and stone formation. After comminuting the stone, part of the HOLC was found in the renal pelvis and the rest of it embedded into renal pelvic wall, making removal of the clip difficult.

In urology, most cases of HOLC migration were documented secondary to laparoscopic or robot-assisted radical prostatectomy. By reviewing the literature, approximate 13 cases of surgical clip migration into the upper urinary tract have been reported (showed in Table 1). Nine of the clinical scenarios happened following minimally invasive partial nephrectomy [2–10]. Two cases occurred after pyeloplasty and another two cases after pyelolithotomy [11–14].

**Table 1:** Previous published studies

Author	Year	No. case	Age	Gender	Initial disease	Initial procedure	Clip types	IHD^1^ (years)	Complications	Diagnostic means	Interventional procedure
Huang	2016	1	71	M	RS^2^	LPL^3^	HOLC	6	RS; hydro	fURS^4^	PNL^5^
Camtosun	2015	1	42	F	RS	LPL	HOLC	2	RS; hydro	URS	URS
Siddharth	2017	1	22	M	UPJO^6^	RPP^7^	HOLC	6	RS	PNL	PNL
Dasgupta	2008	1	41	M	UPJO	LPP^8^	HOLC	Several weeks	RS	PNL	PNL
Ganpule	2020	1	61	F	RCC^9^	LPN^10^	HOLC	8	RS	fURS	fURS
Kiremit	2019	1	48	M	RM^11^	RPN^12^	HOLC	2	US	URS	URS
Shrivastava	2017	1	69	M	AML^13^	LPN	HOLC	3	UTI, calculus	URS	URS
Bayles	2015	1	63	M	RCC	OPN^14^	HOLC	1.5	Clip-strasse	URS	URS
Lee	2014	1	52	M	RCC	RPN	HOLC	2	US; Hydro	fURS	fURS
Fiard	2014	1	67	M	RCC	OPN	HOLC	4 months	US, hydro	URS	URS
Park	2013	1	37	F	RCC	LPN	HOLC	2	US, hydro	URS	URS
Massoud	2011	1	48	F	AML	OPN	APS^15^	9	Flank pain	CT	SP^16^
Miller	2006	1	47	M	RM	LPN	LTC^17^	6 weeks	Pain	CT	SP

^1^Interval of HOLC Discovery

^2^Renal stone

^3^Laparoscopic pyelolithotomy

^4^Flexible ureterorenoscopy

^5^Percutaneous nephrolithotomy

^6^Ureteropelvic junction obstruction

^7^Robot-assisted pyeloplasty

^8^Laparoscopic pyeloplasty

^9^Renal cell carcinoma

^10^Laparoscopic partial nephrectomy

^11^Renal mass

^12^Robot-assisted partial nephrectomy

^13^Angiomyolipoma

^14^Open partial nephrectomy

^15^Autosuture Premium Surgiclip

^16^Spontaneous passage

^17^Lapra-Ty clips

The mechanism of HOLC migration has not been elucidated. However, this particular clinical setting always occurred in the vicinity of anastomosis. One of the possible causes is the excessive suture tension, which makes a chronic continuing erosion of HOLCs [2, 3]. Another reason may be attributed to delayed healing due to chronic kidney disease and diabetes [4]. While in some cases, foreign bodies might erode alongside the sheer stress within our body [15].

Not like the consequence of HOLCs in the lower urinary tract, where they might be passed spontaneously, HOLCs in the upper urinary tract have a propensity to induce complications [16]. The majority of reported patients presented as renal or ureteral stones complicated by hydronephrosis. The narrow lumen of ureter and the curvature of HOLCs in locked position might be attributed to the difficulty of spontaneous passage [5].

As HOLCs are radiolucent and tend to be neglected until a stone has formed. Most reported studies had a misdiagnosis of migrated HOLCs as calculus. Therefore, in a patient with a history of HOLC usage near the collecting system, clip migration and stone formation should be suspected and assessed by CT before treatment planning.

Since HOLCs are SWL-resistant, removal of migrated HOLCs under direct endoscopic visualization is the preferred procedure, including URS, fURS or PNL. In the minority of cases, when HOLCs tightly attached or embedded into the renal parenchyma or the wall of renal pelvis, PNL may be needed [11, 14].

Due to an increase in the report of this rare complication, stabilisation of the suture near the renal pelvis using HOLCs should be avoided. If necessary, such as in minimal access partial nephrectomy, excessive tension on renorrhaphy sutures should be averted to prevent HOLC migration. Instead, in minimal invasive pyelolithotomy or pyeloplasty, knotting should be a better choice.

## Data Availability

Data sharing is not applicable to this article as no datasets were generated or analyzed during the current study.

## Abbreviations

HOLC: Hem-o-Lok clip
CT: Computed tomography
PNL: Percutaneous nephrolithotomy
URS: Ureterorenoscopy
fURS: Flexible ureterorenoscopy
SWL: Shockwave lithotripsy
